# Shaoyao Gancao Decoction for limb dysfunction after fractures around the knee: A protocol for systematic review

**DOI:** 10.1097/MD.0000000000029051

**Published:** 2022-03-18

**Authors:** Xiaohua Shi, Yu Bai, Xiaoming He, Aiguo Wang

**Affiliations:** ^a^ *Department of Minimally invasive Orthopaedics, Zhengzhou Orthopaedic Hospital, Zhengzhou, Henan, China,* ^b^ *Department I of Neck, Shoulder, Waist and Leg pain, Zhengzhou Orthopaedic Hospital, Zhengzhou, Henan, China,* ^c^ *Department of Preclinical, The Third Affiliated Hospital of Henan University of Traditional Chinese Medicine, Zhengzhou, Henan, China.*

**Keywords:** fracture around the knee, limb dysfunction, protocol, Shaoyao Gancao Decoction, systematic review

## Abstract

**Background:**

After reduction and fixation of a fracture around the knee (FAK), excessive injury, improper treatment, soft tissue damage, and blood circulation damage often lead to limb dysfunction, which seriously affects limb rehabilitation and the patient’s quality of life. Shaoyao Gancao Decoction (SGD) is an important means of treatment; however, it is not widely applied because of the lack of evidence about the effectiveness of oral drugs in the treatment of limb dysfunction. This study aimed to evaluate the effects of SGD on patients with limb dysfunction from the perspectives of pain, limb edema, stiffness, as well as physical dysfunction.

**Methods:**

The Chinese and English databases, the Wanfang database, Cochrane Library, China National Knowledge Infrastructure, EMBASE, China BioMedical Literature, PubMed, and Web of Science will be searched from inception to September 30, 2021. Both researchers will select the articles, collect the data and evaluate the quality of the methodology independently using the Cochrane bias risk tool.

**Results:**

High-quality evidence will be obtained to evaluate the beneficial and detrimental effects of SGD on limb dysfunction after FAK, including knee pain, limb edema, stiffness, and physical dysfunction, as well as adverse events.

**Conclusion:**

This study will provide evidence regarding whether SGD is beneficial for treating limb dysfunction after FAK in humans.

**INPLASY registration number:**

INPLASY202210028.

## 1. Introduction

Fractures around the knee (FAK) are common in traffic accidents, often caused by serious direct violence or instantaneous high kinetic energy injury.^[1]^ China has a huge population and is in the primary stage of socialism, hence the growing industry, construction, and transportation have exacerbated the incidence of femoral shaft fractures.^[2]^ Excessive injury, improper treatment, soft tissue damage, and blood circulation damage lead to limb dysfunction, such as limb edema, pain, spasm, rigidity, etc. The affected limb sags and aggravates after activity, and the reduction is not obvious after rest. The condition is repeated, resulting in the slow down and even stagnation of functional exercise progress, which seriously affects limb rehabilitation and the patients’ quality of life.

The commonly used treatment methods are mainly external drugs, functional exercise, and physical therapy, which is difficult to mobilize the internal regulatory function of the human body. In addition, patients with infection, diabetes, and poor skin condition cannot use external drugs. However, there has been no systematic evaluation of therapeutic efficacy as well as safety of oral Shaoyao Gancao Decoction (SGD) for limb dysfunction.^[3,4]^

To address this gap in the literature and provide experts and patients with the latest evidence to evaluate the effectiveness of this therapy and guide clinical practice, we propose to conduct a systemic review to summarize the current evidence regarding the efficacy as well as safety of SGD for limb dysfunction after FAK. The analysis and evaluation of clinical randomized controlled trials (RCTs) involving patients with limb dysfunction will be performed according to the basic principles of evidence-based medicine.

## 2. Methods

This study is registered on the International Platform of Registered Systematic Review and Meta-analysis Protocols (INPLASY), and the ID of the registered study is INPLASY202210028 (https://inplasy.com/inplasy-2022-1-0028/).

### 2.1. Study selection

***2.1.1. Inclusion criteria for study types.*** Only RCTs on SGD for limb dysfunction after FAK, regardless of their publication status or written language, will be included in this study. Laboratory studies, animal experiments, quasi RCTs, reviews as well as case reports will be excluded.

***2.1.2. Inclusion criteria for patients.*** Patients are eligible if they suffered from limb dysfunction after FAK regardless of the severity or duration, gender, ethnicity, or age.

***2.1.3. Inclusion criteria for interventions.*** This review only includes the intervention measures of SGD, including trials comparing SGD with standard treatment and/ or placebo. Trials of SGD combined with other therapies will be included.

***2.1.4. Inclusion criteria for outcomes***

*2.1.4.1. Primary outcomes.* Most patients have lower limb swelling, so measuring the circumference difference between the affected and normal limb has been widely used by clinical researchers as a tool to evaluate limb dysfunction.

*2.1.4.2. Secondary outcomes.* Secondary efficacy endpoints include the HSS knee joint function scale, degree of limb sensory impairment, and related adverse events.^[5]^

### 2.2. Data sources and search strategy

***2.2.1. Electronic sources and search strategy.*** Papers published from their inception to September 30, 2021, in popular databases such as the Wanfang database, Cochrane Library, China National Knowledge Infrastructure, EMBASE, China BioMedical Literature, PubMed, and Web of Science are to be included in this study. The search terms include traditional Chinese medicine, oral traditional Chinese medicine, SGD, supplementary therapy, alternative therapy, herbs therapy, distal femoral fracture, patellar fracture, tibial plateau fracture, fracture around the knee, and randomized controlled trial. Equivalent search terms will be adopted for the Chinese database. All search terms are listed in Table 1, and other searches will be based on these results.^[6]^

**Table 1:** Detailed search strategy for PubMed.

**NO.**	**Search item**
1#	“knee, fracture”[MeSH Terms]
2#	“fracture around the knee”[Title/Abstract]OR ((“femoral condylar fracture”[Title/Abstract] OR “patellar fracture”[Title/Abstrac] OR “tibial plateau fracture”[Title/Abstrac] OR “proximal tibia fracture”[Title/Abstract] OR “distal femoral fracture”[Title/Abstract]
3#	1# OR 2#
4#	“rehabilitation therapies”[MeSH Terms]
5#	“topical drugs”[Title/Abstract] OR “oral drugs”[Title/Abstract] OR “Shaoyao Gancao Decoction”[Title/Abstract] OR “physiotherapy”[Title/Abstract]
6#	4# OR 5#
7#	“randomized controlled trial”[Title/Abstract] OR “controlled clinical trial”[Title/Abstract] OR “Randomized”[Title/Abstract] OR “random allocation”[Title/Abstract] OR “Randomly”[Title/Abstract]
8#	3# AND 6# AND 7#

***2.2.2. Other resources and search strategy.*** The international clinical trial registration platform will also be searched, relevant papers, as well as gray literature, will be identified. Besides, manual searching will be carried out for relevant conference papers and journals.

### 2.3. Data collection and analysis

***2.3.1. Literature screening.*** Literature screening will be conducted independently by 2 researchers. Duplicate literature will be identified and excluded. All literature that fails to meet the established inclusion and exclusion criterion will be excluded. A discrepancy between the 2 researchers will be resolved by referring arbitration to a third researcher.

***2.3.2. Data extraction and management.*** First, data extraction based on a designed standard form will be carried out by 2 independent researchers. The basic information extracted from each study will include clinicodemographic profiles, types of interventions, post-operative complications, and patient outcomes. We will contact corresponding authors in the case of incomplete information.

***2.3.3. Assessment of the risk of bias.*** The quality of included studies will be rated according to 7 parameters as published in the Cochrane Handbook to assess the risk of bias.^[7]^

***2.3.4. Measures of treatment effect.*** For secondary data, a risk ratio with a 95% confidence interval will be used to evaluate the effect. For the continuous data, we will calculate the standardized mean difference (SMDs) with a 95% confidence interval.

***2.3.5. Management of missing data.*** For insufficient or non-existing data, we will contact the author of the original article when necessary to obtain more information or clarification. The existing data will be analyzed only if accurate data is unavailable.

***2.3.6. Heterogeneity assessment.*** The heterogeneity will be assessed using the *I*^2^ value (Higgins *I*-square test), which ranges from 0% to 100% and is divided into 4 levels. Heterogeneity will be considered significant when the *I*^2^ value is more than 50%.

***2.3.7. Subgroup analysis.*** According to the prescription adjustment, dose, course of treatment, and different control groups of SGD, subgroup analysis will be conducted for the study if it shows significant heterogeneity.

***2.3.8. Data synthesis.*** Statistical analysis will be performed using ReMan 5.3. The fixed-effect and random effect models will be established for data synthesis when the *I*^2^ value <50% and >50%, respectively. Subgroup analysis will be performed for significant heterogeneity and an unexplainable clinical pathway.

***2.3.9. Sensitivity analysis.*** When there are enough studies, sensitivity analysis will be conducted to evaluate the robustness of the study based on methodological quality, sample size, and missing data.

***2.3.10. Reporting bias.*** Inverted funnel diagrams will be used to determine the publication bias with regards to circumference difference between the affected and normal limb, HSS knee joint function scale, degree of limb sensory impairment, and related adverse events. If there are >10 trials, a formal statistical test will be conducted for the detection of possible publication bias by investigating funnel plot asymmetry.

## 3. Discussion

After FAK, long-term plaster fixation and braking may result in the patella being between the femoral condyles for a long time, thus limiting patellar sliding up and down. At the same time, fracture, and operation cause hematoma and femoral intermediate muscle injury. The fibrous scar formed by the hematoma adheres to the fibrous scar after femoral intermediate muscle injury and is fixed in front of the femoral shaft. Some people believe that the fiber adhesion between the femoral intermediate muscle and the femoral shaft is the main cause of joint dysfunction. When patients cannot be treated with topical drugs, oral SGD is the main treatment. Although previous studies have reported positive results of acupuncture in the treatment of limb dysfunction of FAK, the supportive evidence remains insufficient.^[8-9]^

The systematic review will provide the latest evidence for the effectiveness as well as safety of SGD in treating limb dysfunction of FAK. These findings will be significant for guiding the treatment of limb dysfunction of FAK in clinical practice, providing a new therapeutic reference for clinicians.

## Author contributions

**Conceptualization:** Xiaohua Shi, Xiaoming He.

**Data curation:** Xiaohua Shi, Xiaoming He.

**Formal analysis:** Xiaohua Shi, Aiguo Wang.

**Funding acquisition:** Yu Bai.

**Investigation:** Xiaohua Shi.

**Methodology:** Xiaohua Shi, Aiguo Wang.

**Project administration:** Xiaohua Shi.

**Resources:** Xiaohua Shi.

**Software:** Xiaohua Shi, Aiguo Wang.

**Validation:** Xiaohua Shi.

**Visualization:** Xiaohua Shi.

**Writing - original draft:** Xiaohua Shi.

**Writing - review & editing:** Xiaohua Shi.

## References

[1] Adeeb M Moran CG. Fractures around the knee. Surg 2003;21: 228-30.

[2] Steele P Bushjoseph C Bachjr B. Management of acute fractures around the knee, ankle, and foot. Clin Fam Pract 2000;3:661-705.

[3] Liu T Zhao X Duan JA Di L Huang Y. Study on anti-inflammatory and analgesic action of total glucosides from Shaoyao Gancao Decoction and its compound mechanism. Tradit Chin Drug Res Clin Pharmacol 2007;6:427-30.

[4] Xiang JL Bao XD. The application of Shaoyao Gancao Decoction in neuromuscular diseases. J Liaoning Univ Tradit Chin Med 2010;1: 208-10.

[5] Narin S Unver B Bakrhan S Bozan O Karatosun V. Cross-cultural adaptation, reliability and validity of the turkish version of the hospital for special surgery (HSS) knee score. Acta Orthop Traumatol Turc 2014;48:241-8. 24901911 10.3944/AOTT.2014.3109

[6] Guyatt G Oxman AD Akl EA, et al. GRADE guidelines: 1. Introduction-GRADE evidence profiles and summary of findings tables. J Clin Epidemiol 2011;64:383-94. 21195583 10.1016/j.jclinepi.2010.04.026

[7] Akl EA Altman DG Aluko P, et al. Cochrane Handbook for Systematic Reviews of Interventions. 2019;Hebei Medicine, 694.

[8] Vun SH Aitken SA Mcqueen MM Courtbrown CM. Adult fracture patterns: epidemiology of fractures around the knee. Orthop Proc 2013; 28:737-8.

[9] Hou W Tang X Zhou Y. Rehabilitation of knee joint functions after surgery of tibia plateau fracture. Hebei Medicine 2011;5:605-7.

